# Effects of sustained i.c.v. infusion of lupus CSF and autoantibodies on behavioral phenotype and neuronal calcium signaling

**DOI:** 10.1186/s40478-017-0473-1

**Published:** 2017-09-07

**Authors:** Minesh Kapadia, Dunja Bijelić, Hui Zhao, Donglai Ma, Ljudmila Stojanovich, Milena Milošević, Pavle Andjus, Boris Šakić

**Affiliations:** 1 Department of Psychiatry and Behavioral Neurosciences, McMaster University, Psychology Building Rm. 303, 1280 Main St., West Hamilton, ON L8S 4K1 Canada; 2 Faculty of Biology, University of Belgrade, Belgrade, Serbia; 3 Department of Pathology and Molecular Medicine, McMaster University, Hamilton, ON Canada; 4 Internal Medicine, “Bezanijska Kosa”, University Medical Center, Belgrade, Serbia

**Keywords:** CNS SLE, Autoimmunity, Cerebrospinal fluid, Brain-reactive autoantibodies, Anti-NMDA receptor, Anti-ribosomal P, Anti-α-tubulin

## Abstract

Systemic lupus erythematosus (SLE) is a potentially fatal autoimmune disease that is often accompanied by brain atrophy and diverse neuropsychiatric manifestations of unknown origin. More recently, it was observed that cerebrospinal fluid (CSF) from patients and lupus-prone mice can be neurotoxic and that acute administration of specific brain-reactive autoantibodies (BRAs) can induce deficits in isolated behavioral tasks. Given the chronic and complex nature of CNS SLE, the current study examines broad behavioral performance and neuronal Ca^2+^ signaling in mice receiving a sustained infusion of cerebrospinal fluid (CSF) from CNS SLE patients and putative BRAs (anti-NR2A, anti-ribosomal P, and anti-α-tubulin). A 2-week intracerebroventricular (i.c.v.) infusion of CSF altered home-cage behavior and induced olfactory dysfunction, excessive immobility in the forced swim test, and perseveration in a learning task. Conversely, sustained administration of purified BRAs produced relatively mild, both inhibitory and stimulatory effects on olfaction, spatial learning/memory, and home-cage behavior. In vitro studies revealed that administration of some CSF samples induces a rapid influx of extracellular Ca^2+^ into murine neurons, an effect that could be partially mimicked with the commercial anti-NR2A antibody and blocked with selective N-methyl-D-aspartate ﻿(NMDA) receptor antagonists. The current findings confirm that the CSF from CNS SLE patients can be neuroactive and support the hypothesis that intrathecal BRAs induce synergistically diverse effects on all domains of behavior. In addition, anti-NMDA receptor antibodies may alter Ca^2+^ homeostasis of central neurons, thus accounting for excitotoxicity and contributing to the heterogeneity of psychiatric manifestations in CNS SLE and other autoantibody-related brain disorders.

**Electronic supplementary material:**

The online version of this article (10.1186/s40478-017-0473-1) contains supplementary material, which is available to authorized users.

## Introduction

Systemic lupus erythematosus (SLE) is a chronic autoimmune/inflammatory disorder accompanied by damage to multiple organs, including the brain [21]. Neurologic and psychiatric (NP) manifestations of varying severity are common, often conferring a grave prognosis and increased mortality rates [40]. Central nervous system (CNS) symptoms can range from focal abnormalities (i.e., seizures, cerebrovascular disease etc.) to diffuse disorders such as anxiety, depression, cognitive impairment, and psychosis [2]. Whereas focal presentations seem to be predominately associated with coagulopathies [103], much less is known about the mechanisms underlying diffuse manifestations. The heterogeneity of neuropsychiatric manifestations points to a complex pathophysiology involving multiple factors that contribute to vasculopathic, glial, and neuronal injury [39].

For many years, autoantibodies reactive to diverse brain antigens (brain-reactive autoantibodies, BRAs) have been attracting considerable attention as terminal factors mediating brain damage and behavioral manifestations in neurological malignancies, seizures, movement disorders, ischemic syndromes, as well as autoimmune diseases including multiple sclerosis and CNS SLE [22, 25]. However, the data from clinical studies are largely correlational in nature and based on the identification of BRAs in the serum [5, 15, 20, 41, 74, 112, 113], cerebrospinal fluid, CSF [6, 13, 35, 50, 117, 118], and post-mortem neuronal tissues of SLE patients [66, 121]. It is not yet clear whether antibodies passively diffuse from the systemic circulation through a breached blood-brain barrier, BBB [1] and/or are synthesized intrathecally during a CNS flare [49, 81, 114] by infiltrating leukocytes [29, 47]. Given the tentative relationship between serum BRAs and NP manifestations [37], autoantibodies in CSF have been proposed as better predictors of CNS involvement [6, 101, 118]. Confirming a cause-effect relationship has proven difficult, partly because the assessment of CNS function in SLE patients can be confounded by peripheral organ damage, opportunistic infections, and treatment with high doses of corticosteroids and cytotoxic agents [12].

More direct evidence supporting a causative role for CSF BRAs stems from experimental studies in murine forms of lupus-like disease [3]. Led in part by the observation of periventricular damage in the spontaneous MRL mouse model [8], two groups concurrently reported that CSF samples from autoimmune mice and CNS SLE patients reduce the viability of murine hippocampal neurons [24, 78]. Across-species cell toxicity was confirmed when CSF samples from behaviorally-impaired mice and another CNS SLE patient were shown to be cytotoxic to a neural stem cell line, neurospheres obtained from lupus-prone and healthy mouse strains, as well as to rat retinal neurons in vivo [92]. Although microfluorometry and electropherograms suggested more than one mechanism of cellular demise, neurotoxicity was primarily accounted for by immunoglobulin G (IgG)-rich fractions of CSF that induced the release of calcium ions (Ca^2+^) from internal stores. Taken together, the results obtained from these studies suggested that antibodies in the CSF bind antigen(s) that are not only shared between immature and differentiated neurons but also conserved amongst mammalian species.

Several autoantigens (expressed centrally and systemically) have been proposed as potential targets of pathogenic BRAs [18, 43, 55, 119]. Among more than 20 BRAs associated with NP manifestations in SLE [119], experimental studies have largely focused on three subgroups. The first is a subset of circulating autoantibodies to double-stranded DNA (anti-dsDNA) that centrally cross-react with the GluN2A and GluN2B subunits of the *N*-methyl-_D_-aspartate (NMDA) receptor [24, 85]. They can access periventricular structures and induce deficits in emotionality and learning/memory when the BBB is chemically-disrupted in healthy mice [52, 66, 67]. When the BBB is bypassed, a single injection of an anti-NMDA receptor antibody into the hippocampus results in excessive neuronal apoptosis [24]. Likewise, acute intracerebroventricular (i.c.v.) injection of anti-ribosomal P antibodies (ARPA) from CNS SLE patients induces “autoimmune depression” characterized by olfactory dysfunction [62, 64] and excessive immobility in the forced swim test [61, 63]. Moreover, intravenous administration of human ARPA impairs memory in otherwise healthy mice following the chemically-induced opening of the BBB [14]. The third subclass includes several antibodies against highly-conserved cytoskeletal proteins including microtubule-associated protein 2 [71, 113], α-tubulin [84], and α-internexin [75]. Although the pathogenic relevance of this last class remains to be explored, a recent study with lupus-prone MRL-lpr mice revealed prominent CSF IgG reactivity to several cytoskeletal proteins [73].

The above evidence supports the hypothesis that BRAs from CSF bind multiple antigens, induce neuronal apoptosis, and ultimately alter behavior. Despite these findings, several gaps in the present knowledge exist. Namely, previous studies involving CNS administration of CSF [24] or BRA [61–64] examined acute effects on behavior, despite the fact that CNS SLE is a chronic condition. In addition, changes in activity-demanding tasks (e.g., water-maze, forced swim test, novel-object recognition) were often interpreted without assessing potentially confounding deficits in spontaneous activity, sensory capacity (e.g., olfaction), motivated behavior, or emotional responsiveness. When antibodies were not delivered directly into the CNS, access of circulating BRAs to the brain parenchyma was dependent on BBB disruption with systemic injections of potent toxins (e.g., lipopolysaccharide) and neuropeptides [14, 52, 66, 67, 75], which per se have profound effects on neuronal metabolism and behavior (reviewed in [65, 80]). Issues related to repeated immunization, lack of comparisons between induced and baseline behavioral performance, reliance on results from short, isolated behavioral tests, unbalanced study designs, and small sample sizes represent additional factors that limit behavioral profiling and increase the odds of data misinterpretation. Using a broad behavioral battery and computerized home-cage monitoring, the present study addresses these concerns by examining the behavioral effects of prolonged i.c.v. administration of CSF from CNS SLE patients and purified BRAs. In order to elucidate the molecular mechanisms underlying BRA-induced neuronal dysfunction, we further explored the modulatory effects of human CSF and purified antibodies on Ca^2+^ metabolism by using differentiated hippocampal neurons and channel-specific blockers.

## Materials and methods

### Study 1: behavioral effects of CSF from CNS SLE patients

A “proof-of-concept” study was undertaken to validate our experimental design by administering undiluted IgG-rich CSF from chronic CNS SLE patients directly into the right lateral ventricle of healthy mice.

#### Human tissue

Given the well-known clinical diversity of CNS SLE, serum and CSF samples from four female outpatients (Lupus Clinic Bezanijska Kosa, University Medical Centre, Belgrade, Serbia) with different psychiatric manifestations were currently used (Table 1). All patients underwent a detailed medical interview and routine physical examination by a qualified rheumatologist, neurologist, and psychiatrist prior to inclusion in the study. Further data regarding various clinical manifestations of the disease, demographic parameters, and laboratory results were obtained from the patients’ medical records. Disease activity was assessed according to the SLEDAI (Systemic Lupus Erythematosus Disease Activity Index) and CNS involvement was confirmed as described earlier [107]. Upon exclusion of common contraindications, CSF was obtained by lumbar puncture of the intravertebral space between the third and fourth lumbar vertebrae. CSF samples from an age-matched, female patient presenting with neuromyelitis optica (NMO) was used as a non-SLE control. Blood-free samples were used exclusively, aliquoted into sterile 100 μl vials, and stored at 4 °C for further analysis.

**Table 1:** Demographic, clinical and autoantibody profile of patients whose CSF samples were used in the study

	NMO	CNS SLE #1	CNS SLE #2	CNS SLE #3	CNS SLE #4
Age	52	59	60	65	49
Sex	Female	Female	Female	Female	Female
SLE duration (years)	0	6	19	9	8
SLEDAI	0	12	12	9	12
Neuropsychiatric manifestations	None	Psychosis Cognitive Dysfunction Vasculitis	Headaches Vasculitis	Depression Cognitive Dysfunction Vasculitis	Anxiety Cerebrovascular Diseases
Serum ANA staining pattern	Negative	Homogenous +	Homogenous +++	Homogenous ++	Speckle +++
Serum anti-AQP4 Positivity	Positive	Negative	Negative	Negative	Negative
Serum anti-dsDNA (g/l)	Negative	163.72	514.44	297.88	25.7
CSF anti-dsDNA (g/l)	Negative	49.46	253.61	77.85	13.46

#### Autoantibody assessment

This analysis included assessment of anti-nuclear antibodies (ANA), anti-dsDNA antibodies and anti-aquaporin4 (AQP4) antibodies in serum and CSF (Table 1).

The degree of ANA positivity was assessed in a fully-automated immunofluorescence assay system (IF Sprinter). Serum samples were diluted 1:80 in sample buffer (pH 7.2), and 30 μl of the diluted serum was pipetted onto HEp-2 cell slides (EUROIMMUN Canada, Mississauga, ON). Slides were incubated for 30 min at room temperature and washed four times with phosphate-buffered saline (PBS)-Tween20 solution. Thirty microliters of 1:100 diluted rabbit anti-mouse IgG-fluorescein isothiocyanate (FITC) conjugate (Sigma-Aldrich, Oakville, ON) were pipetted into each well and left to incubate for 30 min. The slides were washed and sealed with a cover glass following the addition of 10 μl of mounting medium. Nuclear staining patterns were obtained by an unbiased assessor, who scored slides under an LED-fluorescence microscope (EUROStar III, EUROIMMUN).

Levels of anti-dsDNA autoantibodies in serum and CSF were quantified using a fully-automated ELISA analyzer (EUROIMMUN Analyzer I) as previously described [59]. Briefly, 100 μl of each sample (1:50 dilution in PBS buffer) was transferred into a microtiter plate well containing antigen substrate of dsDNA complexed with nucleosomes (Anti-dsDNA-NcX ELISA, EUROIMMUN). Each sample was incubated for 30 min at room temperature and then washed three times with 450 μl of working strength wash buffer. One hundred microliters of 1:2000 diluted rabbit anti-mouse IgG-HRP conjugate (Promega, Madison, WI, USA) were pipetted into each of the microtiter plate wells, left to incubate, and washed to remove unbound HRP enzyme conjugate. Subsequently, 100 μl of 3,3,5,5 tetramethylbenzidine enzyme/substrate solution were pipetted into each well of the microtiter plate and incubated for 20 min at room temperature. One hundred microliters of stop solution were added to each well and the microtiter plate was shaken at 20 Hz for 5 s to ensure a homogeneous distribution of the solution. Optical density was determined at a wavelength of 450 nm and a reference wavelength of 620 nm within 10 min of adding the stop solution. Observed results are expressed as optical densities.

Anti-AQP4 seropositivity was assessed using an immunofluorescence assay employing HEK293 cells transfected with recombinant full-length human AQP4 as described previously [53]. In short, 30 μl of each sample (1:10 dilution in PBS) was applied to BIOCHIP slides using the TITERPLANE technique (EUROIMMUN). After incubation for 30 min at room temperature, the slides were rinsed with PBS-Tween20. Bound IgG were labeled using 30 μl of 1:100 diluted rabbit anti-mouse IgG-FITC conjugate (Sigma-Aldrich) for 30 min and washed as described above. Slides were viewed under an LED-fluorescence microscope (EUROStar III, EUROIMMUN) and sera were classified as positive or negative by an assessor unaware of patients’ diagnoses.

#### Animals

Sixteen ~6-week old CD1 male mice were purchased from Charles River Laboratories (Saint-Constant, QC) and tail-tattooed for identification purposes (AIMS, Inc., Hornell, NY, USA). Males were used exclusively to avoid the confounding effects of estrus cycling on behavioral performance and for ease of surgery (larger cranium/ventricles, less likelihood of anesthetics overdose). Animals were housed four per cage under standard laboratory conditions: reversed light cycle (from 19:00 to 9:00), temperature ~ 22°C, humidity ~62%, ad libitum access to rodent chow and tap water. Assessment of baseline behavioral performance commenced at 10 weeks of age and lasted over a 2-week period. To balance out the groups before the treatment, dependent variables collected to assess baseline performance were reduced by principal component analysis (SPSS v. 20, IBM Corp., Armonk, NY, USA) into a single factorial score. This score was used subsequently to rank each mouse and manually assign it into one of two groups (8 experimental mice receiving CNS SLE CSF vs. eight control mice receiving NMO CSF). The lack of significant differences between selected groups was confirmed using a series of ANOVA tests showing no significant group differences in any dependent measures before the treatments. This group assignment was followed by survival surgery and 2.5-weeks of postoperative behavioral phenotyping.

#### Survival surgery

**Fig. 1:**
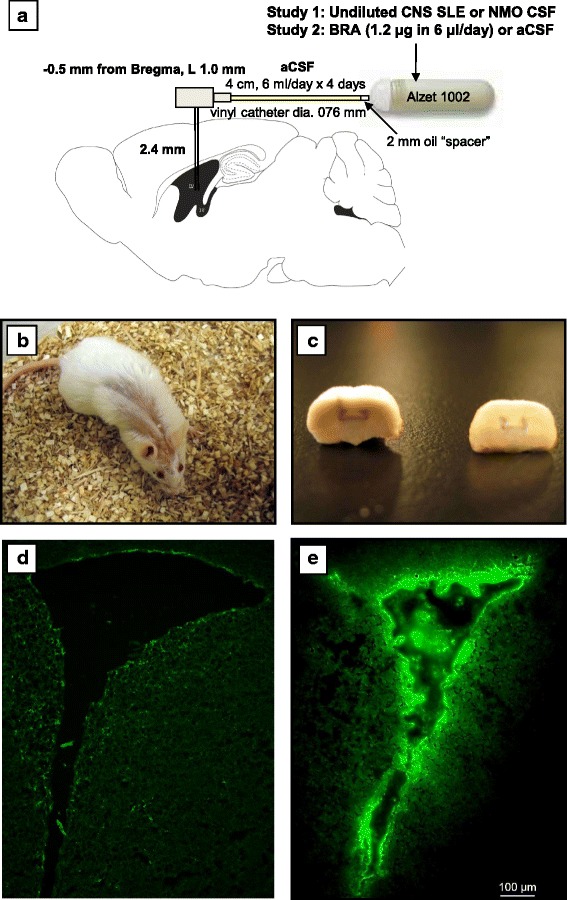
Technical details of survival surgery. **a** All mice underwent unilateral implantation of a sterilized cannula into the right lateral ventricle and subcutaneous implantation of a primed Alzet mini-pump, connected to a cannula via vinyl catheter tubing. Both groups received artificial CSF (aCSF) for 4 days to facilitate postoperative recovery. Hereafter, infusion of the solution of interest was initiated [CNS SLE or control CSF samples in Study 1, purified brain-reactive autoantibodies (BRA) or aCSF in Study 2] and continued for 2 weeks. An oil drop “spacer” was used to prevent mixing of aCSF in the tubing and the experimental solution in the primed pump. **b** An animal moving freely following survival surgery. **c** Histological verification of coordinates obtained by post-mortem injection of Toluidine blue into the vinyl tubing cut at the neck level. **d** Verification of antibody diffusion: control section of the dry ice-fixed contralateral periventricular region after the 2-week i.c.v. administration of CNS SLE serum (**e**) and the same region in another brain showing diffusion gradient in fluorescence when CNS SLE serum was premixed with DyLight 488. *Note:* Images were digitized using an Axioskop 2 Plus microscope with a 5× objective and AxioVision 4.6 software (Carl Zeiss, Inc., CA, USA)

Aseptic survival surgery was performed under ketamine/xylazine anesthetizing cocktail delivered intraperitoneally (0.1 ml/10 g mouse; ketamine: 100 mg/kg; xylazine: 20 mg/kg) and involved i.c.v. implantation of low-cap cannula (model C315GS-4; Plastics One Inc., Roanoke, VA, USA) into the right ventricle (−0.5 mm from Bregma, lateral 1.0 mm), and subcutaneous implantation of primed mini-pumps with a release rate of 0.25 μl/h (model 1002; Alzet, Cupertino, CA, USA). Figure 1a exemplifies technical aspects of the cannula and mini-pump implantation in an adult male mouse. Briefly, each mini-pump was filled with ~100 μl of undiluted CSF from one of four CNS SLE patients and implanted into eight mice (i.e., two mice/CSF sample). The vinyl tube (model C312VT; Plastics One Inc.) connecting the mini-pump to the cannula was pre-loaded with artificial CSF (aCSF) to allow for 4-day postoperative recovery. aCSF was prepared using instructions from Alzet and separated from patient CSF by a 2-mm oil “spacer”. Eight control males were treated in an identical manner, with the exception that the mini-pump was filled with CSF from a patient with NMO. Irrespective of the CSF source, each mini-pump was designed to ensure continuous solution delivery for ~2 weeks in a freely moving mouse (Fig. 1b). Body weight was measured daily before and after the surgery. Mice were sacrificed thereafter and the position of the cannula was verified with Toluidine blue injection into the tubing (Fig. 1c). Antibody diffusion was verified in a pilot study by mixing CNS SLE serum with DyLight 488 amine-reactive fluorescent dye (Thermo Scientific) before the i.c.v. administration. This dye is an NHS ester-activated derivative of high-performance DyLight 488 for fluorescent labeling of antibodies and other proteins. Representative coronal sections of periventricular regions from dry ice-fixed brains are shown in Fig. 1d–e.

#### Behavioral battery

**Fig. 2:**
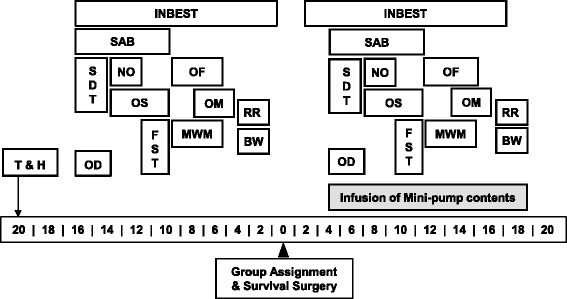
Schematic representation of the experimental design. Prior to testing, all mice were tail-tattooed and habituated to the experimenters. After being assigned to two behaviorally comparable groups, they underwent survival surgery and an identical sequence of tests. The behavioral battery was designed to evolve from less towards more strenuous tasks to mitigate residual stress effects on subsequent tests. *Abbreviations:* T – Tattooing; H – Habituation; INBEST – Integrated Behavioral Station; SAB – Spontaneous Alternation Behavior; SDT – Step-Down Test; NO – Novel Object Test; FST – Forced Swim Test; OF – Open Field Test; MWM – Morris Water Maze; OS – Olfactory Sensitivity; OM – Olfactory Memory; OD – Olfactory Discrimination; BW – Beam-Walking test; RR – Rotarod

Due to technical restrictions on the maximum number of surgeries per day and access to behavioral equipment, a staggered experimental design consisting of three cohorts was used. The protocol sequence included baseline performance, post-surgical performance, and “experimental” performance (i.e., during the infusion of patient CSF, see Fig. 2). In each phase, mice were exposed to a battery of behavioral tests reflective of spontaneous locomotor activity, neurological/sensorimotor function, emotional reactivity and learning capacity that showed discriminatory power in studies with lupus-prone mice [57, 91, 94–98].

The cornerstone of the behavioral phenotyping involved computerized assessment of movements and behavioral acts in an enriched home-cage environment [90]. Each of the eight activity boxes (Integrated behavioral station, ‘INBEST’) comprised of a computer-controlled light stimulus, photocell-controlled lickometers, automated food dispenser, computerized running wheel and shelter (Med Associates Inc., St. Albans, VT, USA). Mice were placed in INBEST for 10 h every other day, permitting continuous collection of measures reflective of spontaneous activity, exploration, and depressive-like behaviors, while minimizing confounding effects induced by inconsistent environmental settings, transportation stress, and repeated handling. Latencies, frequencies, and durations of several behaviors were collected by MedPC IV software (Med Associates Inc.), in parallel with live tracking of ambulation by EthoVision XT 8 software (Noldus Information Technology, Leesburg, VA, USA).

Home-cage phenotyping was supplemented with tests probing neurological function (beam-walking, Rotarod, and olfactory tests), emotionality (step-down, novel object, open field, and forced swim testing), and learning/memory performance (T-maze alternation and Morris water maze).

In the beam-walking test, mice were trained to traverse a narrow beam connecting a brightly-lit starting platform to a dark shelter, as a means to assess fine motor coordination and balance [31, 38, 104]. Following a brief “shaping” procedure, a single run was filmed. Latency to traverse the beam and number of foot slips were scored by an unbiased observed who watched a video clip in slow motion (reviewed in [97]).

A Rotarod (ENV-575 M, Med Associates Inc.) was used to probe balance, muscle strength and acquisition of sensorimotor coordination, as described previously [59, 76]. The Rotarod accelerated from 4 to 40 RPM over 5 min and the latency and speed at fall were recorded automatically.

Olfactory tests were used to assess the ability of mice to detect (sensitivity test), differentiate (discrimination test), and remember scents (memory test). Animals were habituated in an empty, clean cage (45 × 24 × 20 cm) for 8 min and subsequently exposed to a 3 × 3 cm piece of filter paper (Whatman Inc., Piscataway, NJ, USA) scented with 60 μl of an odorant for 2 min. In olfactory sensitivity tests, varying dilutions of peanut butter were tested (diluted to 10^−3^, 10^−4^, 10^−5^ and 10^−6^ in mineral oil) to estimate the detection threshold. Lack of odorant detection was considered when mice spent as much time investigating the odor as the control stimulus (mineral oil alone). The olfactory discrimination test examined the capacity to distinguish different scents using a habituation-dishabituation paradigm [115] with an inter-trial interval of 4 min. Each mouse had four successive exposures to the first odorant (cinnamon, 10^−3^ concentration) before being presented with a dissimilar odorant (paprika, 10^−3^ concentration). An increase in sniffing duration with the novel scent is generally considered indicative of intact discriminatory capacity. Lastly, the olfactory memory test was performed to ascertain the ability of mice to remember a previously presented scent. Mice were exposed to an odorant twice, with 30, 60, 90, and 120 min intervals between the two trials. Odors were randomized, comprising of several commercially available extracts including vanilla, banana, almond, and coconut (10^−3^ concentration; Club House, London, ON). A significant decrease in exploration time upon re-exposure was considered an indication of “olfactory memory”. Experimenters blind to treatment code manually scored duration of sniffing using Observer XT 7.0 (Noldus Information Technology).

The step-down test was performed to measure anxiety-related behavior relating to the readiness of a mouse to descend from an elevated platform (15 × 9 × 9 cm) onto a firm, dark surface in a brightly-lit, unfamiliar room [4, 98]. Latency to step down with all four paws was recorded manually using a stopwatch with a maximum trial duration set at 20 min.

The novel object test, which relies on the approach-avoidance conflict, was used to investigate exploratory drive and anxiety-like response to a novel object [98]. Mice were left to habituate to an arena (45 × 45 × 20 cm) for 5 min before a small cone made of stainless steel was positioned in the center for an additional 5 min. EthoVision XT 8.0 software with a 3-point detection module was used to quantify activity in both “empty” and “full” phases, as well as latency to approach the object, contact frequency, and duration of snout contacts.

The open field is a classical test of exploratory locomotor behavior and emotionality in a spacious environment [94]. In the current study, each mouse was gently lowered along the wall of an enclosed circular arena (diameter = 113 cm) and permitted to explore, uninterrupted for 30 min. The topography of locomotion (center vs. transition vs. thigmotaxic zones), total distance moved, and velocity were automatically analyzed using EthoVision XT 8.0 software.

Increased immobility (floating) of rodents in a situation with no escape has been proposed to reflect a state of “behavioral despair” [87]. In the present study, mice were lowered into a large pool (diameter = 120 cm) filled with water (temperature ~ 25 °C) and left to swim for 10 min. EthoVision XT 8.0 software was used to assess activity. Floating was scored using the built-in mobility module, with “immobility” defined as <5% change in surface area between consecutive samples (rate = 29 samples/s).

Spontaneous alternation behavior (SAB) refers to the intrinsic tendency of mice to alternate their choice of T-maze arms on successive trials. Alternation has been commonly used to assess hippocampal-dependent spatial learning and memory [23]. The discrete-trial procedure was employed for both acquisition and reversal trials, as described previously [10].

Spatial learning and memory were assessed in the Morris water maze (MWM) over the course of 8 days (protocol described in detail [58, 76, 95]). Mice were initially trained in four 2-min “cue trials” with a visible platform (Day 1). On the following 4 days, the platform was hidden and four daily “acquisition trials” from different starting locations were performed. To examine whether a spatial learning strategy was employed, a 2-min “probe trial” was carried out on Day 6 and was followed by three additional “extinction trials”. Subsequently, a cued platform was placed in the opposite quadrant and mice were permitted 2 min to locate it. “Cognitive flexibility” was measured in four “reversal trials” with a hidden platform on Day 8. Measures including latency to find the platform, distance traversed, swimming speed and time spent in the quadrant of interest were obtained with EthoVision XT 8.0.

#### Statistical analysis

Statistical calculations were performed using SPSS 20 software package with the criterion for statistical significance set at *p* ≤ .05 for all group comparisons. Dependent measures were analyzed using Student’s t-test with Treatment (CNS SLE CSF vs. NMO CSF) as a between-group factor. When measures were taken repeatedly (e.g., Intervals, Trials, Concentrations, Days and/or Phases), they were considered within-group factors in analysis of variance (ANOVA) with repeated measures. If significant interactions were detected, Student’s t-test was used for post-hoc comparisons. When appropriate, rates of post-surgery data were taken as a percentage of pre-surgery performance. Rates were calculated using the 1) last mean data point, when pre-operative performance was continuously increasing or decreasing, 2) average, when the pre-surgery behavior was stable or fluctuating. Whenever two treatment groups were compared at one time point, Student’s t-test was performed. Graphs indicate mean values and ± SEM with significant differences of *p* ≤ .05, *p* < .01 and *p* < .001, shown as *, **, and ***, respectively. Graphs represent post-surgery behavioral performance unless specified.

### Study 2: behavioral effects of purified BRAs

An initial attempt to identify pathogenic factors was made by testing effects of three candidate BRAs [52, 62, 63, 66, 67, 84]. Using an experimental design identical to Study 1, the behavioral effects of sustained exposure to anti-NMDA receptor, ARPA, and anti-α-tubulin antibodies were examined.

#### Animals

A total of 72, ~8-week-old CD1 males were tail-tattooed and housed four mice/cage under laboratory conditions described above. Pre-operative behavioral assessment commenced at 11 weeks of age in batches of eight mice. Group assignment was performed as in Study 1. The ranking of factorial scores was followed by manual assignment into six groups (*n* = 12 mice/group) that did not differ a priori in performance. The groups were assigned to an antibody-specific group (anti-NMDA receptor, anti-ribosomal P, or anti-α-tubulin) and corresponding control group (one for each antibody). Consistent with Study 1, post-surgery monitoring of behavioral performance was started immediately and lasted for ~2.5 weeks.

#### Purified antibodies

Commercially available rabbit polyclonal IgG antibodies to NMDA receptor (anti-NR2A, cat. #07-632; Millipore, Billerica, MA, USA), ribosomal P (anti-RPLP0, cat. #NBP1-49979; Novus Biologicals, Oakville, ON), and cytoskeletal protein (anti-α-tubulin, cat.#600-401-880; Rockland Immunochemicals Inc., Limerick, PA, USA) diluted in aCSF were used as surrogates for intrathecal binding of CNS SLE relevant antigens. Prior to dilution in aCSF, antibody solutions were dialyzed as per instructions from Rockland Immunochemicals using Slide-A-Lyzer Dialysis Device (10 K MWCO, cat. # PI69574, Fisher Scientific, Ottawa, ON).

#### Survival surgery

Sterile implantation of low-cap cannulae and primed mini-pumps took place under ketamine/xylazine anesthesia as described above. Each mini-pump was filled with ~20 μg of the experimental antibody and diluted with artificial CSF to achieve an antibody delivery rate of 1.2 μg/day. The cumulative dose over ~2.5 weeks of treatment was calculated to be higher than the dose shown to induce apoptosis of hippocampal neurons in vivo [24]. Control mice were treated in the same way, with the exception that the pump was filled with aCSF. Although an optional design may have employed control IgG, it was shown earlier that even normal IgG in the CSF may induce hyperactivity and depressive-like behavior [82].

#### Behavioral battery

Fifteen days prior to and 15 days after the surgery animals underwent an identical sequence of protocols as in Study 1 (see Fig. 2).

#### Statistical analysis

Statistical analysis and graphical presentation were performed as described in Study 1 above, with Treatment (anti-NMDA receptor, ARPA, or anti-α-tubulin vs. Control) as a between-group factor.

### Study 3: effects of CNS SLE CSF and BRAs on cultured hippocampal neurons

Based on previous studies on the excitotoxic effects of SLE autoantibodies [30, 92], CNS SLE CSF and commercially available BRA were tested for intracellular Ca^2+^ responses in murine neuronal cultures. Compared to our initial study [92], we presently used adult neurons (vs. neural stem cells), a more specific calcium sensitive probe, human CSF (vs. murine), and a better control of extracellular calcium (EGTA vs. EDTA).

#### Primary hippocampal cell culture

Primary hippocampal cell cultures were isolated from newborn (P0) Intor:Swiss mice pups [120]. Once the whole brain was isolated, cerebral hemispheres were peeled back and the meninges surrounding the hippocampi were removed. Thereafter, hippocampi were dissected out and placed into 2 ml tubes filled with cold sterile PBS (in mM: NaCl, 137, KCl, 2.7, Na_2_HPO_4_, 8.1, KH_2_PO_4_, 1.5). Hippocampi from 3 to 5 pups of both sexes were combined. Isolated hippocampi were washed with PBS and enzymatically dissociated with freshly dissolved trypsin from porcine pancreas (1 mg/ml, Sigma-Aldrich) at 37 °C for 10 min with occasional shaking. This was followed by washing and resuspension in plating medium (Neurobasal medium supplemented with 10% fetal bovine serum and 1% GlutaMAX-I, all from Gibco, Invitrogen, and gentamicin 10 μg/ml, Sigma-Aldrich). The suspension was triturated with 1 ml and 200 μl pipette tips and residual debris was allowed to settle down for 1-3 min. Subsequently, the supernatant single-cell suspension was transferred to a new 2 ml tube. Cells were counted and 12,000–15,000 cells were seeded onto 7 mm glass coverslips (#1, Menzel Glasser, Germany), previously coated with poly-D-lysine (10 μg/ml, Sigma-Aldrich). Once the cells adhered (~30 min), the Petri dishes with glass coverslips were filled with 2 ml of growth medium (Neurobasal medium supplemented with 2% B27, 1% GlutaMAX-I, all from Gibco, Invitrogen, and gentamicin 10 μg/ml, Sigma-Aldrich). Neurons were cultivated in a humidified atmosphere of 5% CO2/95% air at 37 °C. Half of the growth medium was changed every other day and cytosine β-D-arabinofuranoside hydrochloride (AraC, Sigma-Aldrich) was added to the growth medium (1 μM on the second day, 3 μM from the fourth day onwards) to suppress glial growth. Primary hippocampal neurons were used in experiments from 7 to 10 days in vitro (DIV).

#### Intracellular calcium imaging and data analysis

Intracellular calcium concentrations were assessed using the cell-permeate acetoxymethyl (AM) ester of Fluo-4 (Fluo-4 AM, Molecular Probes, Eugene, OR, USA). Cells were loaded with 5 μM Fluo-4 AM for 30 min in external solution at 37 °C. Before imaging, cells were washed three times and kept in external solution for an additional 10–15 min at room temperature to allow de-esterification of the dye. Coverslips were then transferred into the recording chamber supplied with 1 ml of working solution (2 mM Ca^2+^ or Ca^2+^-free external solution), placed on an inverted epifluorescent microscope (AxioObserver A1, Carl Zeiss, Oberkochen, Germany) equipped with water, glycerine and oil immersion objective LD LCI Plan-Apochromat 25×/0.8 (Carl Zeiss) and combined with VisiFluor Calcium Ratio Imaging System. The excitation light source was a Xenon Short Arc lamp (Ushio, Japan) combined with a high-speed polychromator system (VisiChrome, Visitron Systems GmbH, Puchheim, Germany). The excitation light (480 nm) and the emission light passed through a FITC filter set (Chroma Technology Inc., VT, USA). Time-lapse images were obtained using the Evolve 512 EMCCD Digital Camera System (Photometrics, Tucson, AZ, USA), every second for 15–45 min via VisiView high-performance imaging software (Visitron Systems). Initially, fluorescence intensities were recorded for 2–5 min to determine the baseline fluorescence (F_0_). Thereafter, 500 μl of diluted CSF from four CNS SLE patients, one control patient with NMO, or commercially available purified antibodies in working solution were applied individually to the imaged cells by customized delivery system, via glass pipettes (0.8 mm inner diameter, positioned ~350 μm away and ~1 mm above the cells, at an angle of 45°) connected to High Speed Solution Exchange System (ALA Scientific Instruments, Farmingdale, NY, USA) with pinch valves and VC3 electronic valve controller. The volume in the recording chamber was kept at ~1 ml by suction from the top of the solution. The response of cells to human CSF/commercial antibodies was recorded for an additional 5 min, followed by constant perfusion of the working solution (washing step), and 50 mM K^+^ was applied to the cells at the end of each experiment to observe their response to depolarization.

The external solution consisted of (in mM): NaCl 140, KCl 5, CaCl_2_ 2, MgCl_2_ 2, D-glucose 10, and HEPES 10, pH 7.4, adjusted with NaOH. For the Ca^2+^-free external solution, CaCl_2_ was omitted and Na_4_EGTA (0.1 mM) was added. For the depolarizing solution, NaCl was lowered to 95 mM, while KCl was increased to 50 mM. For the experiments with a Ca^2+^-free solution, 0.3 mM Na_4_EGTA was added to the diluted human CSF in order to buffer free calcium already present in the CSF [56]. All chemicals (Sigma-Aldrich) were of high purity grade and were dissolved in deionized water (18.2 MΩ). The osmolality of each solution was ~300 mOsM, measured by vapor pressure osmometer (Vapro 5520, Wescor Inc., Logan, UT, USA).

Set of blockers and inhibitors were dissolved per the manufacturer’s instructions and kept in small aliquots at −20 °C. Unless stated otherwise, drugs were purchased from Tocris Bioscience, UK. Tetrodotoxin (TTX), a selective inhibitor of Na^+^ channel conductance, was used in the final concentration of 1 μM to block the generation of action potentials. The cocktail that was used for the blockade of voltage-gated calcium channels (VGCC_block_) consisted of nifedipine (NIF, 10 μM), L-type calcium channel blocker, ω-Conotoxin GVIA (GVIA, 5 μM), a selective blocker of N-type calcium channels, and ω-Agatoxin TK (AGA, 200 nM), a selective blocker of Ca_V_2.1 P/Q-type calcium channels. The inhibitors of glutamate receptor channels (GluR_inh_) used were 6-Cyano-7-nitroquinoxaline-2,3-dione (CNQX, 20 μM), a potent AMPA/kainate receptor antagonist, and DL-2-Amino-5-phosphonopentanoic acid (DL-AP5, 100–200 μM, Sigma-Aldrich), potent and selective NMDA receptor antagonist.

#### Statistical analysis

Raw data were analyzed using SigmaPlot (Systat Software, San Jose, CA, USA), with the criterion for statistical significance set at *p* ≤ .05. The amplitudes of intracellular calcium concentration ([Ca^2+^]_i_) transients, induced by diluted CSF samples or commercially available antibodies, are presented as the mean value of normalized fluorescence intensity ± SEM, with *n* being the number of regions of interests (ROIs). On average, the number of ROIs per frame was 31 ± 3 (mean ± SEM). In pharmacological experiments, amplitudes from the same ROIs were compared before and after drug treatment and analyzed on normalized data using paired t-test. Summary histograms indicate mean of peak amplitudes ± SEM with significant differences of *p* < .001 shown as *. The peaks of the fast and slow component of calcium transients of two effective CSF samples and two effective autoantibodies were compared using Kruskal-Wallis One-Way ANOVA on Ranks with post-hoc Dunn’s Method.

## Results

### CNS SLE CSF infusion induces transient weight loss and alters home-cage behavior

**Fig. 3:**
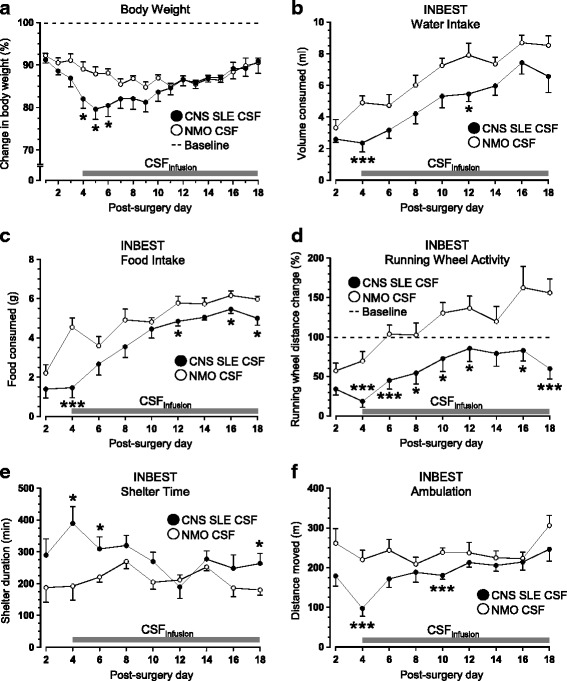
Post-surgery effects of CSF administration on body weight and home-cage behavior in the Integrated Behavioral Station, INBEST. **a** Treatment with lupus CSF was accompanied by a transient, but significant reduction in body weight when CSF infusion began 4 days after pump implantation. In comparison to control mice treated with NMO CSF, experimental animals exposed to CNS SLE CSF (**b**) drank less water and (**c**) consumed less food in INBEST during the 10-h testing period (Days 4 – 18). They also showed (**d**) lower running wheel activity levels, (**e**) prolonged stay in the shelter, and (**f**) decreased ambulation in the home-cage environment (*n* = 8 mice/group)

As expected, a significant drop in postoperative body weight (taken as a percentage of body weight at surgery) was observed in both groups, likely due to the stress induced by general anesthesia and pain. However, mice receiving CSF from lupus patients exhibited a more pronounced reduction in body weight (Day 4: t_14_ = 2.529, *p <* .05; Day 5: t_14_ = 2.841, *p <* .05; Day 6: t_14_ = 2.724, *p <* .05; Fig. 3a). Group differences in weight were transient in nature, only being detected in the initial days of CSF infusion. Administration of CNS SLE CSF led to a prolonged decrease in water (Treatment: F_1,14_ = 9.909, *p* < .01, Fig. 3b) and food consumption (Treatment: F_1,13_ = 28.044, *p* < .001, Fig. 3c). Changes in ingestive behavior were accompanied by impaired running wheel activity, as measured by lower number of rotations (Treatment: F_1,14_ = 28.722, p < .001, Fig. 3d), reduced time spent in the wheel (Treatment: F_1,14_ = 12.672, *p* < .01) and increased time in the shelter (Treatment: F_1,14_ = 22.657, p < .001, Fig. 3e). In addition, infusion of CSF from CNS SLE patients resulted in less overall ambulation (Treatment: F_1,14_ = 12.637, p < .01, Fig. 3f), without affecting movement velocity (Treatment: F_1,14_ = .347, n.s., data not shown).

### CNS SLE CSF infusion impairs olfactory discrimination

**Fig. 4:**
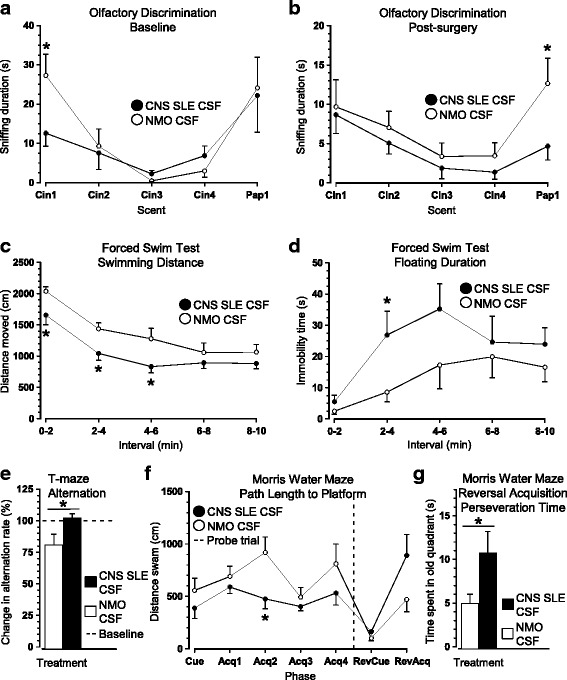
Post-surgical effects of CSF infusion on olfactory function, forced swimming and performance in learning/memory tests. **a** When initially exposed to the olfactory discrimination paradigm during baseline assessment, mice assigned to receive CNS SLE CSF spent significantly more time investigating cinnamon in their first trial but performed comparably to control animals in subsequent exposures. **b** Following surgery, both groups seemed to habituate to repeated exposures to the cinnamon-scented filter paper. However, CNS SLE CSF-treated mice spent significantly less time investigation paprika-laced filter paper in the final (dishabituation) trial. **c** When forced to swim in an empty pool for 10 min, the sustained administration of CSF from CNS SLE patients reduced overall swimming distance and **d** increased floating. **e** Control animals receiving NMO CSF exhibited a significant post-surgery drop in spontaneous alternation rates in the T-maze that was not noted in animals treated with CNS SLE CSF. **f** Despite similar path lengths to locate the cued platform on Day 1, control animals swam longer distances to find a submerged platform on subsequent acquisition trials in the Morris water maze. **g** Even though NMO CSF-treated animals showed relative deficits in acquiring the location of a hidden platform, administration of CNS SLE CSF induced increased perseveration when the platform was re-located and submerged in reversal trials (*n* = 8 mice/group). *Abbreviations:* Cin – Cinnamon; Pap – Paprika; Acq – Acquisition; RevCue – Reversal Cue; RevAcq – Reversal Acquisition

No significant differences in the latency to traverse a narrow beam (Treatment: F_1,14_ = .79, n.s.) or number of beam slips (Treatment: F_1,14_ = .27, n.s.) were noted. Consistent with the results from the beam-walking task, latency to fall from the Rotarod was comparable among mice receiving CNS SLE CSF and those treated with NMO CSF (Treatment: F_1,14_ = 2.489, n.s.). Taken together, these results suggest post-surgical behavioral performance was not confounded by deficits in balance, motor coordination, or muscle strength. However, mice assigned to receive CNS SLE CSF spent significantly more time investigating cinnamon in their first trial (Trial x Treatment: F_1,14_ = 8.771, p < .01, Fig. 4a). However, differences subsided thereafter, as both groups showed comparable habituation to repeated exposures of cinnamon (Treatment: F_1,14_ = .6, n.s.) and similar exploration of a paprika-laced filter paper in the dishabituation trial (Treatment: F_1,14_ = .2, n.s.). When exposed to the same paradigm following mini-pump implantation and CSF infusion, no group differences were noted during cinnamon habituation, although an overall drop in mean sniffing duration was evident. Importantly, mice treated with lupus CSF spent significantly less time investigating the dishabituation odor in comparison to control animals (t_14_ = 2.184, *p* < .05, Fig. 4b). No significant group differences in tests of olfactory sensitivity and short-term olfactory memory were detected (data not shown).

### CNS SLE CSF increases immobility in the forced swim task without altering exploratory and anxiety-like behaviors

Between-group comparisons in the step-down task revealed comparable latencies to step-down from the elevated platform in both treatment groups (t_14_ = .262, n.s.). Similarly, when exposed to the novel object test, groups did not differ in overall locomotion, the topography of movement and exploration of the object (data not shown). They also performed similarly in the open field in terms of distance moved, velocity and time spent in the center and thigmotaxic zones (data not shown). In contrast to dry-land paradigms probing anxiety-like behavior, administration of CNS SLE CSF led to a significant reduction in swimming distance (Treatment: F_1,14_ = 4.870, *p* < .05, Fig. 4c) in the forced swim test. The overall decrease in swimming coincided with increased immobility in CNS SLE CSF-treated animals (2–4 min interval: t_14_ = 2.213, p < .05, Fig. 4d).

### CNS SLE and NMO CSF infusions differentially alter spatial learning and memory

Assessment of SAB revealed that both groups showed comparable alternation rates before surgery (t_14_ = .64, n.s.). However, infusion of control CSF from an NMO patient led to a significant reduction in spontaneous alternation rate post-surgery (Treatment: t_13_ = 2.213, p < .05, Fig. 4e). When tested in the MWM, there were no group differences in the latency to find the cued platform (Treatment: F_1,14_ = .784, n.s.) or the hidden platform over the next 4 days (Treatment: F_1,14_ = 1.968, n.s.). However, control mice displayed longer swim paths to locate the platform over the same acquisition trials (Treatment: F_1,14_ = 4.932, *p* < .05, Fig. 4f). Both groups exhibited similar performance when the platform was removed or moved to the opposite quadrant and made visible. When the same platform was submerged in reversal trials, mice receiving CNS SLE CSF displayed a significantly stronger perseveration response for the quadrant where the platform used to be (Treatment: F_1,14_ = 4.676, p < .05, Fig. 4g). Taken together, CNS SLE CSF did not affect initial response acquisition, yet impaired performance when the task requirements increased in difficulty, likely due to proactive interference. With respect to the SAB data, lower alteration rate further suggests that NMO CSF has detrimental effects on the formation of spatial learning and memory.

### Sustained infusion of anti-NMDA receptor antibodies alters olfactory responsiveness and improves water-maze performance

**Fig. 5:**
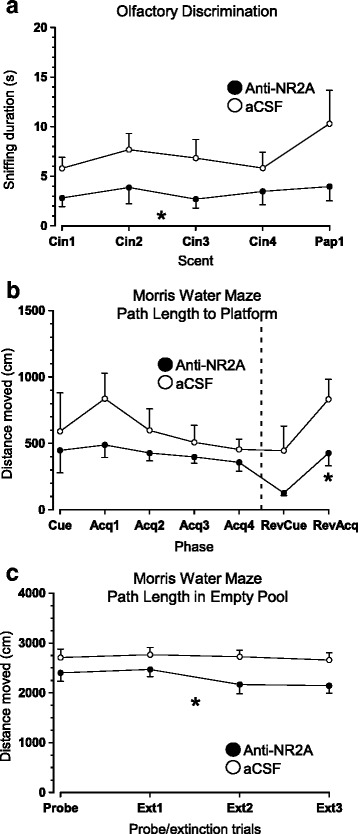
Behavioral effects of anti-NR2A antibody administration. **a** This treatment resulted in a significant reduction in time spent exploring cinnamon and paprika in the olfactory discrimination task. These results suggest that anti-NMDA receptor binding attenuates responsiveness to previously presented scents. **b** Spatial learning/memory assessment in MWM revealed similar performance outcomes in cued and hidden versions of the test. However, when the platform was relocated to the opposite quadrant, mice administered anti-NR2A antibodies exhibited significantly shorter path lengths to locate it. **c** They also swam shorter distances in an empty pool devoid of a platform during probe and extinction trials (*n* = 12 mice/group). *Abbreviations*: Cin – Cinnamon; Pap – Paprika; Acq – Acquisition; RevCue – Reversal Cue; RevAcq – Reversal Acquisition; Ext – Extinction

Behavioral performance between mice receiving anti-NR2A antibodies and controls treated with aCSF was comparable in home-cage setting and in paradigms reflecting neurological function, locomotion, motivated behaviors, and learning/memory (data not shown). Task-specific differences, however, were evident in the olfactory tasks and the MWM. Namely, mice treated with anti-NR2A antibodies spent significantly less time sniffing cinnamon, during the four habituation exposures, compared to mice that were administered aCSF only (Treatment: F_1,22_ = 5.217, *p* < .05; Fig. 5a). When comparing the exploration of the dishabituation scent, there was a trend for aCSF-treated animals to spend more time with paprika (t_22_ = 1.730, *p* = 0.098). These results suggest that anti-NMDA receptor treatment alters responsiveness to previously-exposed scents, without necessarily affecting habituation or discriminatory capacity.

Two outliers (one from each treatment group) that consistently failed to find the platform were excluded from MWM analyses. Performance in the cued and acquisition phases of the test was comparable between groups (data not shown). When the task was made more complex in reversal trials, mice that received the anti-NR2A antibodies exhibited shorter path lengths to locate the newly-positioned hidden platform (Treatment: F_1,20 *=*_ 5.161, *p* < .05; Fig. 5b). They also swam shorter distances in an empty pool devoid of a platform during probe and extinction trials (Treatment: F_1,20 *=*_ 4.611, *p* < .05; Fig. 5c). Taken together, sustained anti-NMDA receptor administration seemingly improved “cognitive flexibility” when reaching a relocated, hidden platform.

### Sustained infusion of ARPA alters metabolic demands and search strategy

**Fig. 6:**
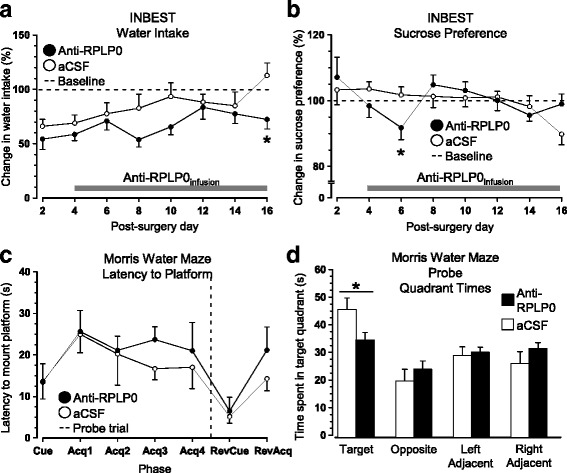
Behavioral effects of sustained anti-RPLP0 antibody administration. **a** Administration of anti-RPLP0 antibodies into the brains of healthy mice led to a transient decrease in water consumption that was prominent on the last day of INBEST testing. **b** These mice also displayed a transient, but significant reduction in preference for 8% sucrose solution, as compared to their baseline performance. **c** When exposed to the MWM, no group differences were detected in the latency to locate a cued or hidden platform in acquisition and reversal trials. **d** However, ARPA-treated mice displayed poorer recall when the platform was removed from the pool during the probe trial (n = 12 mice/group). *Note:* Eight animals had to be excluded from the analysis of the probe and extinction trials due to a technical error with video-tracking software. *Abbreviations*: INBEST – Integrated Behavioral Station; Acq – Acquisition; RevCue – Reversal Cue; RevAcq – Reversal Acquisition

Like the results obtained with anti-NR2A, infusion of anti-RPLP0 antibodies did not influence overall performance in most behavioral paradigms tested. Although, group differences were not observed in general measures of activity in INBEST (distance moved, velocity, running wheel), mice treated with anti-RPLP0 antibodies displayed alterations in water intake (Day x Treatment: F_7,154_ = 2.195, *p* < .05, Fig. 6a **)** and sucrose preference **(**Treatment: F_1,22_ = 5.647, p < .05; Fig. 6b). In the MWM, there were no differences in the latency to locate a cued or hidden platform during acquisition and reversal trials (Fig. 6c), but anti-RPLP0-treated mice exhibited poorer spatial bias for the target quadrant when the platform was removed from the pool in the probe trial (t_14_ = 2.224, p < .05, Fig. 6d). The results suggest that sustained ARPA infusion into healthy mouse brains alters metabolic demands and search strategies. Both treatment groups performed comparably in all other behavioral paradigms (data not shown).

### Sustained infusion of anti-α-tubulin antibodies stimulates spontaneous behavior and reversal learning

**Fig. 7:**
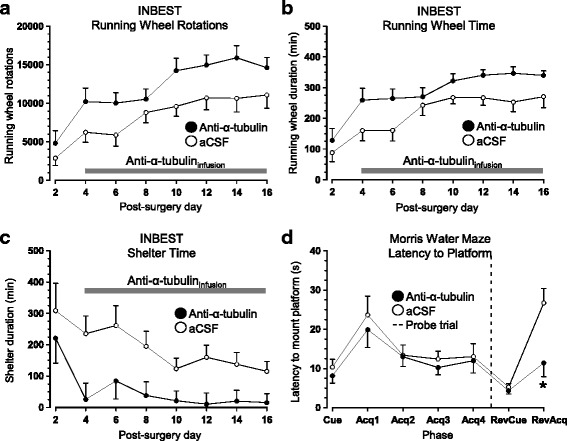
Behavioral effects of sustained anti-α-tubulin antibody administration. **a** Prolonged infusion of anti-α-tubulin antibodies increased post-surgical home-cage activity, as measured by increased number of running wheel rotations, **b** more time spent in the running wheel, and **c** less time spent in the shelter. **d** In the MWM, mice had similar latencies to locate cued and hidden platforms. However, relocation of the platform in reversal acquisition trials resulted in significantly shorter escape latencies in mice treated with anti-cytoskeletal antibodies. Together with home-cage behavior, these results suggest that sustained administration of anti-α-tubulin antibodies has a stimulatory effect on certain behaviors (n = 12 mice/group). *Abbreviations*: INBEST – Integrated Behavioral Station; Acq – Acquisition; RevCue – Reversal Cue; RevAcq – Reversal Acquisition

Post-surgery monitoring of home-cage behavior revealed that infusion of anti-α-tubulin antibodies increased running wheel activity, as measured by wheel rotations (Treatment: F_1,19_ = 5.163, p < .05, Fig. 7a) and time spent in the running wheel (Treatment: F_1,19_ = 5.518, p < .05, Fig. 7b). Coinciding with higher running wheel duration, mice treated with anti-α-tubulin antibodies also spent less time in the shelter (Treatment: F_1,19_ = 9.431, p < .05, Fig. 7c). Other INBEST measures related to ingestive behaviors, ambulation, and velocity were similar in both groups (data not shown). In the MWM, mice from both groups performed comparably on cued, acquisition and probe trials (Fig. 7d). However, when the hidden platform was placed in the opposite quadrant, anti-α-tubulin antibody administration correlated with a shorter escape latency (Treatment: F_1,95_ = 4.405, p < .05, Fig. 7d). Taken together, the results are consistent with the notion that sustained administration of anti-α-tubulin antibodies had a stimulatory effect on certain aspects of behavioral performance. Mice treated with anti-α-tubulin performed comparably to animals receiving only aCSF in all other respects (data not shown).

### CSF samples from CNS SLE patients induce diverse intracellular calcium transients

**Fig. 8:**
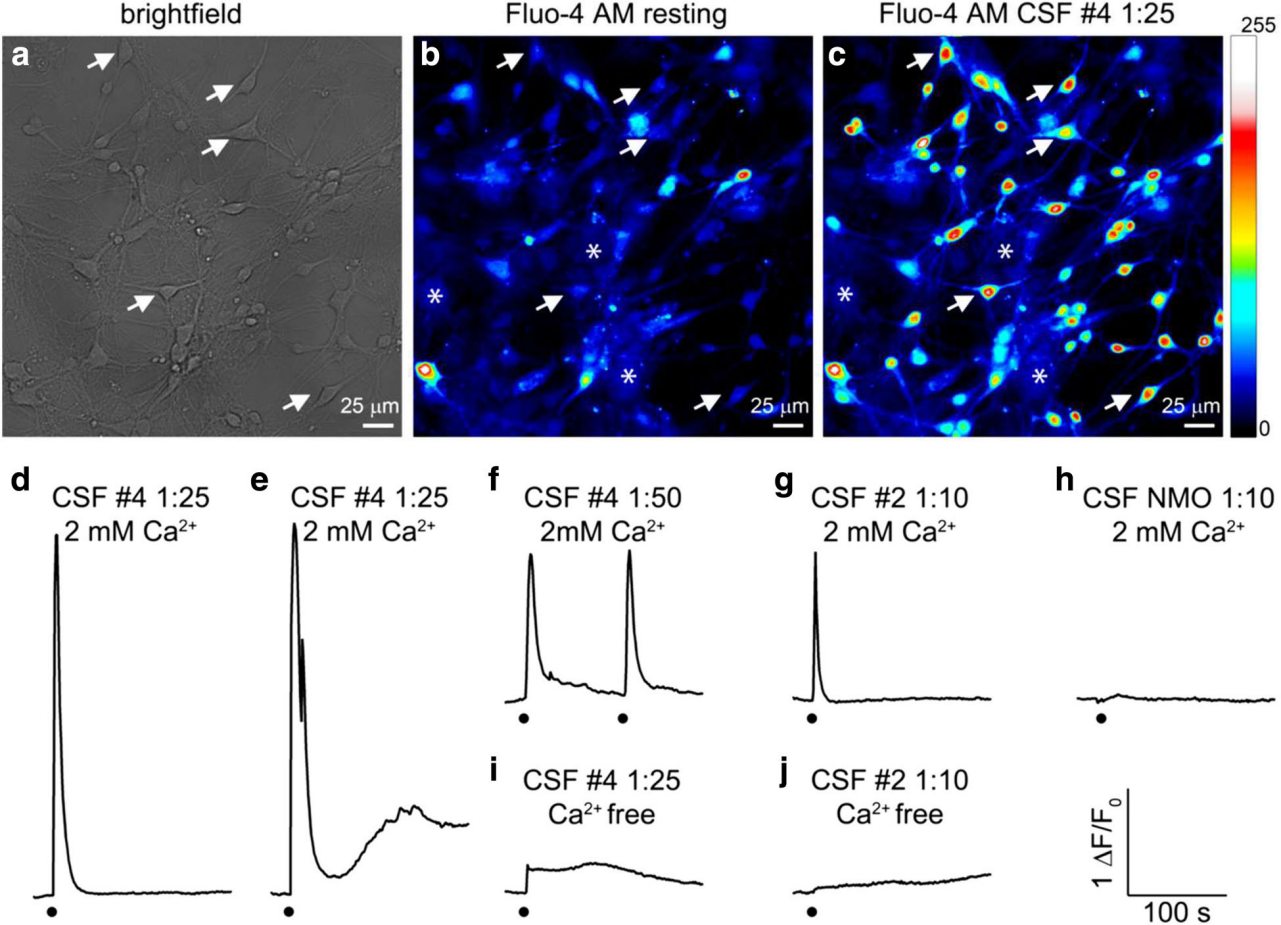
Examples of CSF-induced intracellular calcium transients in cultured hippocampal neurons. **a** Brightfield (transmitted light) image of cultured hippocampal neurons. **b** Fluorescent image of hippocampal neurons loaded with 5 μM Fluo-4 AM, in color-coded intensity scale (0–255, right) before the application of CSF. **c** An example of intracellular calcium peak response to CNS SLE CSF #4, 3 s after application. Scale bars for (**a–c**) are shown in lower right corner and cell bodies of neurons and astrocytes are indicated by arrows and asterisks, respectively. **d–j** Representative traces of intracellular calcium responses (normalized fluorescence intensity) of hippocampal neurons challenged by CSF from CNS SLE or NMO patients. CSF application is indicated by a black dot below the trace. CSF origin, dilution, as well as the presence/absence of external calcium is indicated above the traces. In an external solution with 2 mM Ca^2+^, 1:25 CNS SLE CSF #4 induced two types of responses, with (**d**) only fast or with (**e**) fast and late slow components. **f** The same sample at 1:50 dilution was also able to increase the cytosolic calcium concentration in the repeated application, to the same level as the first response. **g** 1:10 CNS SLE CSF #2 induced a fast calcium transient in 2 mM Ca^2+^. **h** Disease control NMO CSF in 1:10 dilution did not influence the intracellular calcium concentration in 2 mM Ca^2+^ external solution. **i** In a Ca^2+^-free external solution, 1:25 CNS SLE CSF #4 did not induce the fast transient, and **j** no calcium response could be detected after application of 1:10 CNS SLE CSF #2. *Note:* Calibration for (**d–j**) is shown in the lower right corner

Analysis consisted of extracting average fluorescence intensities from ROIs that were drawn around the neuronal bodies. Hippocampal cultures at 7-10 DIV exhibited typical pyramidal neuronal bodies and many interconnected branches forming a network (Fig. 8a). Astrocytes were also present in the cultures, despite treatment with AraC. However, due to their flat morphology, they were not easily visible in the brightfield images. Conversely, fluorescent images of Fluo-4 AM loaded cells showed both neurons and astrocytes (Fig. 8b–c), with astrocytes exhibiting spontaneous calcium activity that (in some cases) interfered with signals from neuronal bodies. Hence, such cases were left out from further analysis. In addition to ROIs that encompassed neuronal bodies, five ROIs were extracted from the background, at each time point of the experiment. The background correction was done by subtracting averaged background fluorescence from each ROI at every time point. Under resting conditions, the signal intensity from the calcium indicator did not exceed 10% of the dynamic range of acquisition (Fig. 8b). Cells with high resting intensity were excluded from further analysis. Upon stimulation (peak of the CNS SLE CSF shown in Fig. 8c), the intensity of fluorescence increased linearly with the increase in the intracellular calcium concentration ([Ca^2+^]_i_), allowing us to evaluate the changes in [Ca^2+^]_I_ by normalizing the change in fluorescent intensity to the resting level (ΔF/F_0_) in the dynamic range of the acquisition.

Table 2 summarizes the effects of CSF samples from four CNS SLE and one NMO patient on cytosolic Ca^2+^ homeostasis in cultured hippocampal neurons. Two of the four CSF samples from CNS SLE patients induced transient changes in [Ca^2+^]_i_. Ten times diluted CSF from patient #4 increased the [Ca^2+^]_i_ above the dynamical range of the acquisition, so further tests were done with higher dilutions. At a dilution of 1:25, this sample induced two types of responses: a fast [Ca^2+^]_i_ transient (Fig. 8d **;** time-lapse video clip enclosed as Additional file 1: Video S1) and a late, slow component that was seen in some neurons (Fig. 8e). Testing a higher dilution (1:50) revealed a dose dependent effect in which the amplitude of [Ca^2+^]_i_ transient gradually decreased with the dilution, from 3.00 ± 0.18 (1:25 dilution, *n* = 51), to 1.23 ± 0.08 (1:50 dilution, n = 51). Moreover, the effect of CNS SLE CSF #4 was repeatable, as the second stimulation, applied 100 s after the first stimulus (both in 1:50 dilution), induced the [Ca^2+^]_i_ transient of the same shape and amplitude (Fig. 8f). CSF from CNS SLE patient #2 was effective in the 1:10 dilution exclusively ([Ca^2+^]_i_ amplitude = 1.29 ± 0.18, *n* = 31) and exhibited only one type of response with fast [Ca^2+^]_i_ transient (Fig. 8g). CSF from the NMO patient did not have any effect on cytosolic calcium (Fig. 8h).

**Table 2:** Four CNS SLE and one NMO CSF sample characterized by their ability to induce intracellular calcium responses in cultured hippocampal neurons

CSF Dilution	NMO	CNS SLE #1	CNS SLE #2	CNS SLE #3	CNS SLE #4
1:10	–	–	+	–	+++
1:25	n.t.	n.t.	–	n.t.	++
1:50	n.t.	n.t.	n.t.	n.t.	+

Calcium transient detected (+) with the number of symbols corresponding to the transient relative amplitude; not detected (−); not tested (n.t)



**Additional file 1: Video S1.** Time-lapse video showing calcium transients in a culture of adult hippocampal neurons exposed to CNS SLE CSF#4 (1:25 dilution) at 0 s time, washed around 5 min, and then challenged with K^+^ solution at the 8-min mark. (WMV 11519 kb)


### Ca^2+^-free solution abolishes the effect of CNS SLE CSF

To identify the source of calcium ions that contribute to CNS SLE CSF-induced [Ca^2+^]_i_ transients, further experiments were conducted in Ca^2+^-free external solution. External calcium was proven to be the major source of intracellular transients, as Ca^2+^-free external solution substantially reduced the effect of 1:25 CNS SLE CSF #4 to 0.32 ± 0.04 (*n* = 26, Fig. 8i) and completely abolished the effect of CNS SLE CSF #2 (*n* = 17, Fig. 8j).

### Voltage-gated calcium channels and glutamate receptors are implicated in CNS SLE CSF-induced Ca^2+^transients

**Fig. 9:**
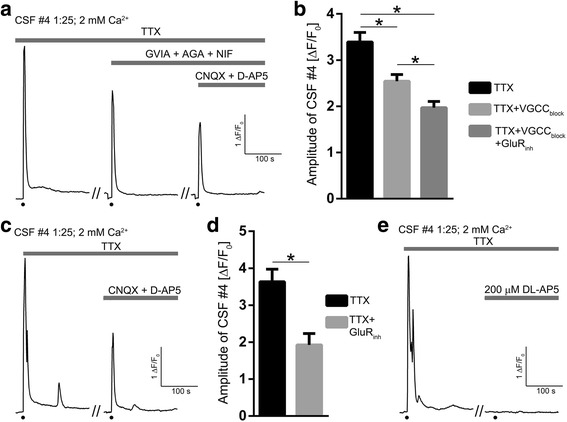
Effects of blockers of voltage-gated calcium channels and inhibitors of ionotropic glutamate receptors on CNS SLE CSF #4 - induced calcium transients. **a** A representative trace of normalized fluorescence with gradually decreasing amplitudes following differential drug treatments to block action potentials (TTX), voltage-gated calcium channels (VGCC_block_) and AMPA/kainate and NMDA receptors (GluR_inh_). **b** Summary histogram of peak amplitudes confirming that blockade of voltage-gated and ionotropic receptors decreases the amplitude of the control response. **c** Representative trace and **d** summary histogram of CNS SLE CSF #4 - induced calcium transients showing that GluR_inh_ drugs alone lower the amplitude of the control response. **e** NMDA receptor blockade with 200 μM DL-AP5 completely abolished the control response, suggesting a predominant role for NMDA receptors in CNS SLE CSF-induced calcium transients. The addition of CSF in (**a**, **c**, **e**) is indicated by black dots below the trace. Drugs used are indicated by the gray timelines above the trace and calibration within the trace on the right. * *p* < .001, two-tailed paired t-test. *Abbreviations*: TTX – Tetrodotoxin; VGCC_block_ – GVIA + AGA + NIF; GVIA – ω-conotoxin GVIA; AGA – ω-agatoxin TK; NIF – Nifedipine; GluR_inh_ – CNQX + D-AP5; CNQX – 6-Cyano-7-nitroquinoxaline-2,3-dione; D-AP5: D-2-Amino-5-phosphonopentanoic acid; DL-AP5 – DL-2-Amino-5-phosphonopentanoic acid

To elucidate specific receptor mechanisms, we employed blockade of the action potential using 1 μM TTX. All experiments were performed with 1:25 CNS SLE CSF #4 and amplitudes from the same ROIs were compared in different pharmacological treatments and analyzed on normalized data. A representative trace of gradually decreasing amplitude with the addition of drug combinations is shown in Fig. 9a. The application of 5 μM GVIA, 0.2 μM AGA, and 10 μM NIF (to block the voltage-gated calcium channels - VGCC_block_) reduced the amplitude of the control response from 3.39 ± 0.21 to 2.54 ± 0.15 (t_43_ = 9.07, *p* < .001), suggesting that voltage-gated receptors take part in CNS SLE CSF-induced calcium transients (Fig. 9b). Similarly, the addition of 20 μM CNQX and 100 μM D-AP5 (to inhibit AMPA / kainate and NMDA receptors - GluR_inh_) further reduced the amplitude to 1.97 ± 0.13, and this reduction in amplitude was significant vs. both control response (t_43_ = 13.16, p < .001), and VGCC_block_ (t_43_ = 18.56, *p* < .001) implying a role for ionotropic receptors (Fig. 9b). Next, we examined whether ionotropic or voltage-gated calcium channels underlie CNS SLE-induced calcium transients. GluR_inh_ drugs were solely able to lower the amplitude of the control response from 3.63 ± 0.34 to 1.92 ± 0.31 (t_15_ = 15.95, *p* < .001, representative trace Fig. 9c, summary histogram Fig. 9d). The averaged amplitudes of the two control responses did not differ significantly and no difference could be detected between the inhibition of only ionotropic (GluR_inh_) and both ionotropic and voltage-gated receptors (VGCC_block_ + GluR_inh_). Finally, to distinguish between AMPA/kainate and NMDA receptor involvement, 200 μM DL-AP5 (NMDA receptor blocker in higher concentration) was applied. This blockage completely abolished the control response in every tested ROI (*n* = 23, see example in Fig. 9e), suggesting a predominant role for NMDA receptors in CNS SLE CSF-induced Ca^2+^ transients.

### Commercially available BRAs fail to mimic CSF-induced Ca^2+^transients

**Fig. 10:**
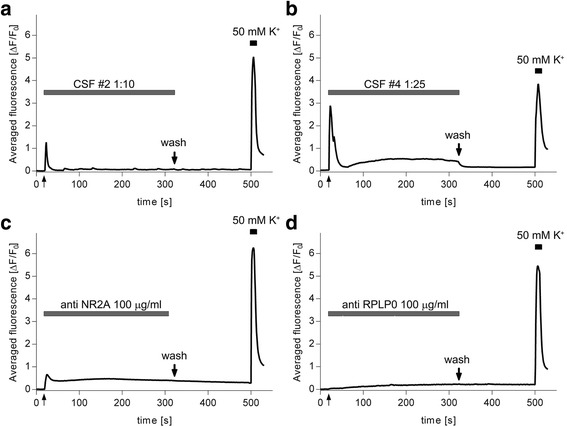
Comparison of averaged intracellular calcium transients evoked by effective CNS SLE CSF and commercially available antibodies in time. **a** Trace illustrating that CNS SLE CSF #2 diluted to 1:10 evoked only a fast transient that peaked 3 s after CSF application. **b** The calcium response to 1:25 CNS SLE CSF #4 exhibited a fast transient after 3 s that was followed by a slower component measured 3 min after application and a return to basal levels after wash. **c** Anti-NR2A evoked a fast calcium transient that peaked at 6 s and a slow component that peaked after 3 min and could not be washed. **d** Anti-RPLP0 failed to induce a fast transient but provoked a slow and sustained rise of [Ca^2+^]_i_ 3 min after application. The application of CSF/antibodies in (**a**–**d**) is indicated by an upward arrow, duration by the gray line above the trace, wash by a thick downward arrow, and depolarizing 5 s pulse (50 mM K^+^) by a black strip above the trace. The origin and dilution of CSF samples, as well as the antibodies and concentration used, are indicated above the gray line

To narrow down immunoglobulins accounting for neuroactive effects of autoimmune CSF, we assessed Ca^2+^ transients after administration of commercially available anti-NR2A, anti-RPLP0, and anti-α-tubulin antibodies. We also tested a high concentration of albumin since CNS SLE CSF #4 showed ~500 μg/ml of this protein. Figure 10 compares averaged responses of effective CSF samples and BRA followed by wash after 5 min and a depolarizing test (50 mM K^+^) at the end of experiments. The averaged shape of 1:10 CNS SLE CSF #2 calcium response exhibited only a fast transient that peaked 3 s after CSF application, with an amplitude of 1.28 ± 0.18 (*n* = 31, Fig. 10a). Conversely, the 1:25 CNS SLE CSF #4 – induced calcium response exhibited not only the fast transient that peaked 3 s after application (2.89 ± 0.18), but also a slower component with an amplitude of 0.53 ± 0.09 (measured 3 min after application), as well as a returning of the signal to a basal level after wash (*n* = 51, Fig. 10b). In contrast to diluted CNS SLE CSF samples, anti-NR2A antibody induced a fast calcium transient (0.66 ± 0.10, *n* = 38) that peaked at 6 s, showed a slow component (0.46 ± 0.03) 3 min after and remained even after wash (Fig. 10c). Anti-RPLP0 antibody provoked only a slow rise of [Ca^2+^]_i,_ with an amplitude of 0.19 ± 0.02 (*n* = 13) 3 min after application, that also could not be washed (Fig. 10d). Pairwise comparisons of the fast calcium transients among treatments (both CNS SLE CSF and anti-NR2A) differed significantly (Kruskal Wallis, H2 = 60.028, p < .001; Dunn’s post-hoc tests, *p* < .05). On the other hand, all pairwise comparisons of the slow components among treatments (CNS SLE #4, anti-NR2A, anti-RPLP0) revealed a significant difference only between the two commercial autoantibodies (Kruskal Wallis, H2 = 12.414, p < .05; Dunn’s post-hoc tests, p < .05). Neither anti-α-tubulin nor albumin induced any perturbations in [Ca^2+^]_i_ levels (data not shown). Taken together, the results obtained suggest that the effects of CNS SLE CSF are heterogeneous and complex, and cannot be mimicked by an individual antibody.

## Discussion

Given the chronic and complex nature of CNS SLE, the current study examines in vivo and in vitro effects of sustained i.c.v. infusion of human CSF samples and putative autoantibodies on broad behavioral performance and neuronal calcium signaling. Significant differences were found in several behavioral domains, but the sustained administration of putative BRAs could not recapitulate the broad spectrum of behavioral manifestations detectable in the spontaneous murine MRL model of lupus, or in healthy mice treated with undiluted CNS SLE CSF. Moreover, we showed that CSF from two out of four lupus patients induced neuronal activation through Ca^2+^ influx, which in one case seemed to be mediated via the NMDA receptor system. However, anti-NR2A and anti-RPLP0 antibodies could not induce [Ca^2+^]_i_ responses comparable to the effects of patient CSF. Although the importance of other BRAs is not excluded, the present results suggest that the synergistic effect of multiple BRAs underlies altered neuronal metabolism and behavioral dysfunction in CNS SLE.

The results obtained with CNS SLE CSF are largely consistent with previous reports detailing CSF neurotoxicity in human and murine forms of lupus [24, 78, 92]. Whereas these earlier studies focused on cellular effects, our results suggest that BRA-rich CSF promotes an assortment of impairments in metabolic demands, emotional reactivity, olfactory function, and motivated behaviors. In this regard, the functional profile of animals receiving autoimmune CSF mimics several aspects of the so-called “autoimmunity-associated behavioral syndrome” that lupus-prone MRL-lpr mice develop [93, 108]. Characteristic behavioral deficits in this substrain include confined exploration in the vicinity of their “home-base” [96, 98], reduced responsiveness to palatable stimulation [9, 91], response perseveration in spatial learning tasks [10, 46, 96], excessive immobility when forced to swim [98], and altered olfactory function [57, 60]. The two latter impairments, operationally termed “autoimmune depression” by Katzav and colleagues, have also been noted in another murine model of CNS SLE induced through the acute i.c.v. infusion of ARPA [61–64]. Yet, the results from the current study do not entirely support such a relationship. In particular, while the transient decrease in sucrose preference could be reflective of depressive-like behavior, anti-RPLP0 administration also produced a reduction in water intake, thus raising the possibility that these mice also have altered metabolic demands. Instead, the finding of spatial reference memory deficits in anti-RPLP0-treated mice is more in line with recent studies demonstrating that passive transfer of ARPA induces learning impairments in otherwise healthy animals [14, 61]. The results are intriguing given that we could not reproduce similar deficits using anti-NMDA receptor antibodies, as others have noted in paradigms of learning and memory [26, 66, 67]. Our findings, however, are consistent with several reports that have been unable to replicate the association between anti-NR2 antibodies and behavioral impairments in SLE patients [42, 45, 68–70, 86, 106] and MRL-lpr mice [77]. Relative improvements in acquiring the novel escape location following anti-NR2A exposure suggest that anti-NMDA receptor antibodies may have a stimulatory role at certain concentrations and under certain experimental conditions. Such results would be in line with neuroprotective effects elicited by anti-NR1/NR2 antibodies in experimental models of stroke, epilepsy and neuropathic pain [27, 111]. By the same account, anti-α-tubulin antibodies also seemed to have a stimulatory effect, one that was more robust and detected on multiple performance measures. Although the results may be analogous to the known effects of anti-NMDA receptor antibodies, they are largely inconsistent with clinical associations [84] and experimental evidence with another cytoskeletal autoantibody [75].

We have previously documented an abrupt cytotoxic effect of CSF from a deceased CNS SLE patient on a neural stem cell line [92]. The effect was corroborated by a microfluorimetrically measured (calcein-sensitive) rise of intracellular ions that was not dependent on extracellular calcium, but instead on the inhibitors of intracellular Ca^2+^ store channels. By employing a more specific Ca^2+^-sensitive probe, the results herein confirm the induction of intracellular ionic misbalance by CNS SLE CSF with a clear role of Ca^2+^ signaling in this process. Contrary to our previous study, the CSF effect in the present study was fully dependent on extracellular Ca^2+^. This discrepancy may be attributable to the different neuronal cell types employed (primary neurons in culture versus a neuronal stem cell line), as well as to the application of CSF from different patients. Other groups have also tested the effects of serum BRA on [Ca^2+^]_i_ homeostasis, including ARPA in primary cortical neurons [79, 102] and anti-GluN2 antibodies on GluN1/N2a-transfected HEK 293 cells [36]. In all these studies, BRA induced Ca^2+^ entry into cells through membrane receptors, but only anti-GluN2 antibodies were shown to induce Ca^2+^ entry by affecting the binding capacity of zinc and not the channel itself [36]. The latter study used the same BRA concentration, as we did in the present study. However, Matus and colleagues obtained well-defined transients (in 67% neurons) with just 0.1 μg/ml ARPA. In our hands, 100 μg/ml anti-RPLP0 caused a modest, albeit sustained and irreversible [Ca^2+^]_i_ rise, which was not observed in previous studies [79, 102]. This difference could be explained by different sources of antibodies – purified from patient sera [79, 102] versus commercially available in the present study.

Notably, the effect of CNS SLE CSF on [Ca^2+^]_i_ could be induced with a 1:50 dilution. However, these in vitro experiments also demonstrate significant heterogeneity, which is typical of the overall CNS SLE symptomatology (see Table 1). This could be seen in terms of concentration-dependent effects (1:50 CSF #4 induced a response comparable to 1:10 CSF #2) and in terms of the response shape (e.g., [Ca^2+^]_i_ spike alone, or followed by a late dampened prolonged transient). The effect was shown to be predominantly attributed to postsynaptic NMDA receptors, as it could be completely abolished by DL-AP5. Nevertheless, the effect of VGCC cannot be completely excluded via Ca^2+^-dependent presynaptic release or by removing voltage-dependent Mg block from NMDA receptor. One may hypothesize that the effect of CNS SLE CSF is mediated directly by IgG Fab’2 fragments, unrelated to complement activation, as shown for DNA/NMDA receptor-reactive antibodies [30].

Our pharmacological dissection experiments suggest that the CSF effect is mediated mainly via glutamate receptors. Nevertheless, inhibitors of AMPA and NMDA receptors, regardless of VGCC block, abolished merely 50% of the [Ca^2+^]_i_ transient amplitude. Using only the NMDA receptor inhibitor DL-AP5 could completely abolish the response to CNS SLE CSF, albeit at a rather high concentration (200 μM, at which the blocker might have been unselective). Our cell cultures also contained astrocytes, which may interact with neurons and their Ca^2+^ signaling via gliotransmission [19, 44]. Given the importance of concentration–dependent effects [79], a more elaborate dose-dependent analysis with a larger CSF volume would be justified in a future study.

It is noteworthy that the in vitro tested commercially available antibodies against NR2A or RPLP0 had a slight (although sustained) effect on neuronal [Ca^2+^]_i_. However, the concentration used (100 μg/ml) was above the maximal IgG titer in the tested undiluted CNS SLE CSF sample (see Table 2). Contrary to the sustained [Ca^2+^]_i_ phase present in some responses to CNS SLE CSF #4, the continuous response to BRA was not washable. This may indicate an immune complex (antigen-antibody) reaction, while the response of neurons to diluted CSF may be less specific, yet well defined. On the other hand, the IgG fraction isolated from sera of CNS SLE patients could also induce a similar, well-defined [Ca^2+^]_i_ responses in rat neuronal cultures (ms in preparation), reminiscent of the effect of whole CSF herein. In addition, the effect of BRA could become apparent through synergy with glutamate release and action as in the case of NMDA receptor-reactive antibodies in lupus [30]. However, the physiological significance of the autoantibody-induced sustained [Ca^2+^]_i_ response phase remains to be elucidated.

The mechanisms underlying the heterogeneity of responses induced by CNS SLE CSF and the diminished potency of purified commercially available antibodies to do the same requires further consideration. Firstly, CSF samples from CNS SLE patients are likely to contain a plethora of antibodies with differing reactivates to neuronal tissues. Indeed, our own analysis of CNS SLE CSF (ms in preparation) revealed elevations in several antibodies that were not tested in the current study, but have been linked to CNS SLE manifestations including anti-dsDNA [24, 33, 66, 85], anti-cardiolipin [41, 74, 83, 99] and anti-PR3 antibodies [100]. Therefore, it is plausible that the relatively pronounced effects elicited by undiluted CNS SLE CSF samples are mediated by these BRA classes. Given the abundance of autoantibodies in lupus [116], an alternative, but equally likely possibility involves the induction of behavioral manifestations by as of yet undetectable BRA in the CSF. While some of the autoantibodies in CSF may ultimately represent epiphenomena, other classes may have potent effects across a wide range of concentrations, even in negligible amounts. In the current study, we used a cumulative dose of 20 μg over ~2 weeks, with a daily delivery rate of 1.2 μg/per day. The final dose was significantly larger than the amount used by DeGiorgio and colleagues to induce local neuronal loss with an anti-NMDA receptor antibody [24], but considerably smaller than the dose used by Katzav and colleagues to induce behavioral changes using ARPA [61–63]. Although beyond the scope of this report, one may further hypothesize that different concentrations of a particular antibody, much like the binding of different antigen targets, can produce starkly dissimilar effects. Recent findings show that low concentrations of anti-NMDA receptor antibodies selectively amplify NMDA-mediated synaptic signaling, but promote excitotoxic cell death via mitochondrial dysfunction at high concentrations [30]. Relatively low concentrations may help explain why we noted stimulatory roles for both anti-NR2A and anti-α-tubulin antibodies in certain paradigms. Another factor to consider is the source of the antibodies. Whereas the current study used polyclonal antibodies against singular antigens in isolation, previous studies involving direct CNS administration have employed purified human antibodies against multiple antigens and epitopes [24, 61–63]. Binding of regions outside of major immunoreactive domains in human NMDA receptor, ribosomal P and α-tubulin proteins could be another factor at play. Therefore, a possible combined effect of these antibodies against multiple epitopes on other cross-reactive antigens might be the reason for the pathological effects seen surrounding more non-specific BRAs. The data may further serve as a potential model to explain discrete CNS symptoms along a wide spectrum, some that are caused by a transient effect on neuronal functioning and others caused by permanent neuronal damage [51].

The interpretation of the present findings is complicated by the observation that CSF from an NMO patient had no effect on [Ca^2+^]_I_, but seemingly impaired performance in spatial learning and memory tasks. NMO is an inflammatory demyelinating disorder of the CNS that is primarily characterized by the presence of autoantibodies to AQP4 in serum [72] and CSF [109]. The target antigen is an integral membrane protein that forms the most abundant water channel in the CNS, but anti-AQP4 antibodies have been specifically implicated in BBB dysfunction, altered glutamate homeostasis, and induction of necrotic cell death in the optic nerve and the spinal cord [54]. Passive transfer of purified patient NMO IgG fractions, as well as recombinant human anti-AQP4 IgG, produces key aspects of NMO-like CNS lesion pathology, including loss of AQP4 expression, myelin breakdown, axonal injury, extensive inflammatory cell infiltration, astrocyte depletion, and neuronal cytotoxicity in a complement-dependent manner [7, 11, 88]. More recently, intrathecal administration of anti-AQP4 was found to elicit similar, but reversible histopathological changes independently of complement activation and immune cell infiltration [34]. One may surmise that the impaired performance in both the T-maze and water maze reflects anti-AQP4-induced vision deficits. However, this possibility seems unlikely given that the mice perform comparably to CNS SLE CSF-treated animals in cued platform trials. Given the high expression of AQP4 messenger RNA by neurons in periventricular structures of the rodent brain [110], a more likely explanation may involve preferential binding of anti-AQP4 IgG to regions like the hippocampus that are implicated in the acquisition, consolidation, storage, and retrieval of spatial information in the water-maze and related paradigms [58]. Nevertheless, further study of this selective effect of NMO CSF on behavioral performance in spatial learning tasks is necessary, particularly because NMO CSF had no effect on intracellular Ca^2+^. This would support the hypothesis that NMO-specific IgG acts through a mechanism distinct from a [Ca^2+^]_i_ signaling pathway [34, 109] that is dependent on the activation of complement (not present in cell cultures) to promote pathology [48, 89]. In summary, the current study supports a neuropathogenic role for BRAs in the CSF of some CNS SLE patients. In addition, it provides a conceptual basis for the identification of novel diagnostic markers [28] and targets in pharmacotherapy of CNS SLE. Identification of other pathogenic autoantibodies and concentration-dependent effects may also help to shorten the list of candidate BRAs in lupus and advance our understanding of autoimmune mechanisms in demyelinating diseases [32], autoimmune encephalopathies [22], autism spectrum disorder [16, 17], and schizophrenia [105].

## Conclusions

The sustained infusion of autoantibody-rich cerebrospinal fluid from CNS SLE patients into the brains of healthy animals induces alterations in home-cage behavior, olfactory dysfunction, depressive-like behavior and perseveration in a learning task. The administration of putative BRAs in a similar manner produces relatively mild, both inhibitory and stimulatory effects on olfaction, spatial learning/memory, and home-cage behavior. In vitro studies reveal that some CSF samples induce a rapid influx of extracellular Ca^2+^ into murine neurons via the glutamatergic system.
